# Risk factors for maternal mortality in the west of Iran: a nested case-control study

**DOI:** 10.4178/epih/e2014028

**Published:** 2014-11-08

**Authors:** Jalal Poorolajal, Behnaz Alafchi, Roya Najafi Vosoogh, Sahar Hamzeh, Masoomeh Ghahramani

**Affiliations:** 1 Modeling of Noncommunicable Disease Research Center, Department of Epidemiology and Biostatistics, School of Public Health, Hamadan University of Medical Sciences, Hamadan, Iran; 2 Department of Epidemiology and Biostatistics, School of Public Health, Hamadan University of Medical Sciences, Hamadan, Iran; 3 Department of Family Planning, Vice-Chancellor of Health Services, Hamadan University of Medical Sciences, Hamadan, Iran

**Keywords:** Maternal mortality, Maternal death, Risk factors, Case-control studies, Iran

## Abstract

**OBJECTIVES:**

With a gradual decline in maternal mortality in recent years in Iran, this study was conducted to identify the remaining risk factors for maternal death.

**METHODS:**

This 8-year nested case-control study was conducted in Hamadan Province, in the west of Iran, from April 2006 to March 2014. It included 185 women (37 cases and 148 controls). All maternal deaths that occurred during the study period were considered cases. For every case, four women with a live birth were selected as controls from the same area and date. Conditional logistic regression analysis was performed and the odds ratio (OR) and its 95% confidence interval (CI) were obtained for each risk factor.

**RESULTS:**

The majority of cases were aged 20-34 years, died in hospital, and lived in urban areas. The most common causes of death were bleeding, systemic disease, infection, and pre-eclampsia. The OR estimate of maternal death was 8.48 (95% CI=1.26-56.99) for advanced maternal age (≥35 years); 2.10 (95% CI=0.07-65.43) for underweight and 10.99 (95% CI=1.65-73.22) for overweight or obese women compared to those with normal weight; 1.56 (95% CI=1.08-2.25) for every unit increase in gravidity compared to those with one gravidity; 1.73 (95% CI=0.34-8.88) for preterm labors compared to term labors; and 17.54 (95% CI= 2.71-113.42) for women with systemic diseases.

**CONCLUSIONS:**

According to our results, advanced maternal age, abnormal body mass index, multiple gravidity, preterm labor, and systemic disease were the main risk factors for maternal death. However, more evidence based on large cohort studies in different settings is required to confirm our results.

## INTRODUCTION

Maternal mortality is an important public health indicator because it represents a measure of the overall effectiveness of healthcare delivery systems [1]. Maternal health refers to the health of women during pregnancy, childbirth, and the postpartum period [2]. The fifth goal of the United Nations Millennium Development Goal (MDG 5) focused on improving maternal health to reduce maternal mortality by three quarters by 2015 [3]. Unfortunately, the global rates of change suggest that only 16 countries will achieve the MDG 5 by 2015 [4]. Despite the fact that most maternal deaths are preventable, every day 800 women die from preventable causes related to pregnancy and childbirth [5]. About 45% of postpartum deaths occur within 24 hours [6]. The main direct causes of maternal morbidity and mortality include hemorrhage, infection, high blood pressure, unsafe abortion, and obstructed labor [2].

Although the leading causes of maternal death are well known, there is still a large disparity in the maternal mortality ratio (MMR) between low- and high-income countries. Almost all maternal deaths (99%) occur in developing countries. In 2013, the MMR was 230 per 100,000 live births in developing countries vs. 16 per 100,000 live births in developed countries. In addition to the between-country variation in maternal mortality, there is a large disparity within countries; for example, between women with high and low income and between women living in rural and urban areas [5-8].

The Islamic Republic of Iran has implemented several effective policies relating to population control and family planning that have resulted in a significant reduction in the total fertility and crude birth rates over the past three decades [9,10]. As the result of population control, Iran has achieved a dramatic reduction in the MMR, which has fallen to a rate comparable with developed countries [9]. The MMR has declined dramatically in Iran during the three decades since the Islamic Revolution. In 1980, the MMR was 101 per 100,000 live births in Iran. This fell to 64 per 100,000 in 1990, and continued to decline to 35 per 100,000 in 2000 and 28 per 100,000 in 2008 [11].

Maternal mortality as an MDG 5 target needs attention. With the gradual decline in maternal mortality in recent years in Iran, it is important to identify the remaining potential risk factors for maternal death. There are several direct or indirect socio-demographic indicators that affect maternal mortality [12]. These indicators vary across countries and regions [2]. Until reliable information on these indicators is collected, it is difficult to design effective intervention strategies to progress towards achieving the fifth MDG. Accordingly, this 8-year nested case-control study was designed and conducted to accurately investigate the main causes of maternal death in Hamadan Province, in the west of Iran.

## MATERIALS AND METHODS

The Research Committee of Hamadan University of Medical Sciences approved this study. This 8-year nested case-control study was conducted in Hamadan Province, in the west of Iran, from April 2006 to March 2014. The study population included women of reproductive age with at least one pregnancy who had healthcare records in the rural or urban health centers of the province. Hamadan Province includes nine cities and a population of 1,819,777 people. In this province, there are 163 health centers (93 in rural and 70 in urban areas) and 579 health houses [13]. During the 8-year study period, 37 maternal deaths occurred. The deaths occurred in different parts of the province in both urban and rural areas.

The cases were women who died from causes related to pregnancy or childbirth. The maternal deaths were reviewed and confirmed by the Provincial Maternal Death Expert Committee according to the 10th revision of the International Classification of Diseases codes. During the study period, 37 cases were found. Since the number of cases was small, four controls were selected for every case from the same study population. The controls were selected from women with a live birth. Controls were individually matched to cases by death date and the area of residence. For this purpose, we randomly selected four women who had given live birth in the same place and at the same time as each maternal death.

Data were extracted from primary healthcare records available at the urban or rural health centers using a checklist of items according to the context of the health records. The checklist included data on mothers’ age, gestational age (preterm vs. term), body mass index (BMI), gravidity, the type of childbirth (normal vaginal delivery vs. cesarean section), the primary person managing the delivery (midwife vs. obstetrician), and the reason for death (bleeding, pre-eclampsia, infection, and systemic disease). Systemic diseases included chronic heart failure, diabetes, high blood pressure, renal disease, and thyroid impairment. Preterm labor, or so-called premature labor, was defined as a gestational age of less than 37 completed weeks [14]. BMI, the weight in kilograms divided by the square of the height in meters (kg/m^2^), was classified according to the recommendation of the World Health Organization [15] as follows: a BMI less than 18.5 kg/m^2^ was underweight, a BMI greater than or equal to 18.5 kg/m^2^ was normal weight, a BMI greater than or equal to 25 kg/m^2^ was overweight, and a BMI greater than or equal to 30 kg/m^2^ was obese.

Conditional logistic regression analysis was performed to assess the effect of various risk factors on maternal death. In order to control for the effect of potential confounding factors, a backward stepwise-adjusted analysis was performed to fit the data well and to exclude unnecessary variables from the model. For this purpose, we started with a full model and then excluded variables one at a time, while checking via the likelihood ratio test to determine whether the reduced model or the full model fitted the data significantly well. Crude and adjusted odds ratios (ORs) were reported to address the association between maternal death and each of the associated predictors. Alpha was set at 0.05. Analysis was performed using Stata version 11 (StataCorp, College Station, TX, USA).

## RESULTS

During the study period 37 maternal deaths occurred. Cases were compared with 148 controls (four controls for every case). The mean (standard deviation [SD]) age of cases and controls was 30.19 (6.03) and 26.92 (6.15) years, respectively (p=0.004).

The characteristics of the cases are given in Table 1. The majority of the cases were aged 20-34 years, died in hospital, and were urban residents. The most common causes of death were bleeding, systemic disease, infection, and pre-eclampsia.

Results from the simple and multiple conditional logistic regression analysis of the predictors of maternal death are given in Table 2. Compared to women aged younger than 34 years, the OR estimate of maternal death for women aged equal to or older than 35 years was 2.45 (95% CI=1.01-5.99). After controlling for other variables, this OR estimate of maternal death increased to 8.48 (95% CI=1.26- 56.99).

There was a direct association between maternal death and abnormal BMI. Compared to women with normal weight, the OR estimate of maternal death was 1.86 (95% CI=0.42-8.14) in underweight women and 3.63 (95% CI=1.33-9.89) in overweight or obese women. These OR estimates increased to 2.10 (95% CI=0.07-65.43) and 10.99 (95% CI=1.65-73.22), respectively, when adjusted for other variables.

Compared to normal vaginal delivery, cesarean section increased the risk of maternal death by 1.83 (95% CI=0.82- 4.09). However, the adjusted analysis revealed an inverse association. Compared to normal delivery, the adjusted OR estimate of maternal death for birth by cesarean section was 0.92 (95% CI= 0.22-3.89).

The OR of maternal death was 1.86 (95% CI=0.82-4.19) in women whose infants were delivered by obstetricians compared to those delivered by midwives. This variable was emitted from the adjusted model because of goodness of fit.

There was a direct association between gravidity and maternal death, so that the higher the gravidity, the higher the risk of maternal death. The OR estimate of maternal death was 1.56 (95% CI =1.08-2.25) per unit increase in gravidity. However, this variable was emitted from the adjusted model because of goodness of fit.

There was a strong association between gestational age and maternal death. The OR estimate of maternal death was 4.30 (95% CI=1.82-10.17) for preterm labors compared to term labors. However, this OR estimate decreased to 1.73 (95% CI= 0.34- 8.88) when adjusted for other variables.

Systemic diseases were the strongest risk factor for maternal mortality. The OR estimate of maternal death in those who had a systemic disease was 6.88 (95% CI=2.61-18.17) compared to those without any chronic disease. After controlling for the potential confounding effect of other variables, the association became much stronger and reached 17.54 (95% CI=2.71-113.42).

## DISCUSSION

The results of this study indicate that maternal death is associated with a number of predictors including advanced maternal age, gravidity, advanced gestational age, underlying systemic disease, and abnormal BMI.

About 56.8% of the maternal deaths occurred in urban areas (compared to 43.2% in rural areas). Given that 70% of the Iranian population lives in urban areas, these data reveal the disparity in risk for maternal death between urban and rural areas. Because of the extensive nationwide health network in Iran, access to primary healthcare services in rural areas is even better than in urban areas (active care in rural areas vs. passive care in urban areas), but access to secondary and tertiary health services, such as technical and specialized antenatal care, is not as good in rural areas, especially in remote villages, as it is in urban areas. Failure to access adequate health services in emergency situations may be the reason for the greater proportional risk of maternal death in the rural areas. Furthermore, there are many obstacles that prevent women from receiving or seeking care during pregnancy and childbirth, including poverty, distance, lack of transportation, prolonged transportation, lack of information, inadequate services, and cultural practices [5,16].

According to the crude OR estimates, the risk of maternal death was unexpectedly higher in women who were assisted by an obstetrician than those who were assisted by a midwife. The reason may be that those women who were assisted by an obstetrician might have had a higher baseline risk than women who were assisted by a midwife. Generally, some women experience serious health problems during pregnancy. These complications make the pregnancy a high-risk pregnancy that requires emergency medical intervention [17]. Therefore, women who receive and seek specialized health services may be at higher risk of pregnancy complications.

According to our results, the leading cause of death was bleeding, followed by systemic disease, pre-eclampsia, and infection. About 31.4% of the deaths were for other reasons. Ramos et al. [18] performed a multicenter population-based study to identify the risk factors in 121 maternal deaths. According to the results of that study, the most common causes of maternal death were abortion complications (27.4%), hemorrhage (22.1%), infection/sepsis (9.5%), hypertensive disorders (8.4%), and other causes (32.6%).

Based on our findings, preterm labor was a strong predictor of maternal death. Previous studies have confirmed this result. Conde-Agudelo et al. [19] reported that premature rupture of membranes can increase the risk of maternal death by 1.72 (95% CI=1.53-1.93).

We initially found that cesarean section increased the risk of maternal mortality nearly two-fold. However, adjusted OR estimates revealed an inverse relationship between cesarean section and maternal death. According to multiple analyses, those who underwent cesarean section had a lower risk of death compared to those who had a normal vaginal delivery. This indicates that women who underwent cesarean section may require emergency medical interventions but despite the higher baseline risk had a lower risk of death because of obstetric assistance. Furthermore, some women underwent elective cesarean sections with no baseline risk. There was not enough information in the health records to separate emergency cesareans from elective ones. However, previous studies have reported a direct association between cesarean section and maternal mortality and morbidity. Liu et al. [20] assessed maternal mortality and morbidity associated with 46,766 planned cesarean deliveries vs. 292,420 planned vaginal deliveries and concluded that the planned cesarean group had increased postpartum complications such as cardiac arrest, wound hematoma, hysterectomy, infection, venous thromboembolism, and hemorrhage. In addition, Deneux-Tharaux et al. [21] performed a population-based case-control study to assess the risk of maternal death associated with cesarean in comparison with vaginal delivery and concluded that the risk of postpartum death was 3.6 times higher after cesarean than after vaginal delivery.

Our data revealed that the risk of maternal death increased with gravidity, in agreement with Conde-Agudelo et al. [22], who examined the association between multiple gravidity and maternal outcomes and concluded that multiple gravidity significantly increased the risk of maternal morbidity and mortality.

The main limitation of this study was the small number of maternal deaths. In order to overcome this limitation, we extended the duration of study to eight years and enrolled as many cases of maternal death as possible. However, no more than 37 maternal deaths were found during the study period. On the other hand, we increased the number of controls for every case to improve the statistical power. Despite this limitation, the current study reveals the major risk factors for maternal death in the target population in a middle-income country. The results may be helpful for policymakers who plan preventive programs and prioritize risk factors in order to reduce the burden of maternal mortality and morbidity.

In conclusion, several predictors affect maternal mortality and morbidity in the west of Iran, including advanced maternal age, multiple gravidity, gestational age older than 35 years, underlying systemic disease, and abnormal BMI. However, more evidence based on large cohort studies in different settings is required to confirm these results.

## Figures and Tables

**Table 1.** Characteristics of mothers (n=37) who died from causes related to pregnancy and cildbirth

Variable	n (%)
Age (yr)	
15-19	1 (2.7)
20-34	26 (70.3)
35-46	10 (27.0)
Location of death	
Hospital	32 (91.4)
Home	2 (2.9)
On way to hospital	1 (5.7)
Region of residence	
Urban	21 (56.8)
Rural	16 (43.2)
Causes of death	
Bleeding	13 (37.1)
Infection	3 (8.6)
Pre-eclampsia	3 (8.6)
Systemic disease^1^	5 (14.3)
Other^2^	11 (31.4)

1 Systemic diseases included chronic heart failure, diabetes, high blood pressure, renal disease, and thyroid impairment;

2 Other reasons included pulmonary embolism, brain hemorrhage, acute myocardial infarction, unsafe abortion, and aortic dissection.

**Table 2.** Odds ratio (OR) estimates of maternal mortality by demographic and clinical characteristics of the study population using logistic regression model (pseudo R^2^=0.5698)

Variable	Controls (n=148)	Cases (n=37)	Unadjusted OR (95% CI)	p-value	Adjusted OR (95% CI)^1^	p-value
Maternal age at delivery (yr)						
<34	126	27	1.00		1.00	
≥35	20	10	2.45 (1.01-5.99)	0.049	8.48 (1.26-56.99)	0.03
Body mass index (kg/m^2^)						
Normal weight (18.5-24.9)	85	11	1.00		1.00	
Underweight (<18.5)	12	3	1.86 (0.42-8.14)	0.41	2.10 (0.07-65.43)	0.67
Overweight/obese (≥25.0)	34	14	3.63 (1.33-9.89)	0.01	10.99 (1.65-73.22)	0.01
Type of delivery						
Normal vaginal delivery	82	12	1.00		1.00	0.91
Cesarean section	64	18	1.83 (0.82-4.09)	0.14	0.92 (0.22-3.89)	
Person who managed delivery						
Midwife	80	11	1.00			
Obstetrician	66	18	1.86 (0.82-4.19)	0.14	-	-
Gravidity						
First	71	13	1.00			
Per unit increase in gravidity	75	24	1.56 (1.08-2.25)	0.02	-	-
Gestational age (wk)						
Term (37-42)	117	19	1.00		1.00	
Preterm (< 37)	25	15	4.30 (1.82-10.17)	0.001	1.73 (0.34-8.88)	0.51
Systemic disease^2^						
No	121	17	1.00		1.00	
Yes	22	17	6.88 (2.61-18.17)	0.001	17.54 (2.71-113.42)	0.003

CI, confidence interval.

1 OR adjusted for other variables in the table;

2 Systemic diseases included heart disease, diabetes, high blood pressure, renal disease, and thyroid impairment.

## References

[1] King JC (2012). Maternal mortality in the United States--why is it important and what are we doing about it?. Semin Perinatol.

[2] World Health Organization Maternal health. http://www.who.int/topics/maternal_health/en/.

[3] United Nations Millennium development goals and beyond 2015. http://www.un.org/millenniumgoals/bkgd.shtml.

[4] Kassebaum NJ, Bertozzi-Villa A, Coggeshall MS, Shackelford KA, Steiner C, Heuton KR (2014). Global, regional, and national levels and causes of maternal mortality during 1990-2013: a systematic analysis for the Global Burden of Disease Study 2013. Lancet.

[5] World Health Organization (2014). Maternal mortality. http://www.who.int/mediacentre/factsheets/fs348/en/.

[6] Nour NM (2008). An introduction to maternal mortality. Rev Obstet Gynecol.

[7] Women and Health Alliance International Maternal health. http://www.waha-international.org/?health-topics&gclid=CI7Uw6qg0r8CFZJr7AodtxMAkw.

[8] Amirian H, Roshanaei G, Poorolajal J, Esmailnasab N, Moradi G (2014). Analyzing socioeconomic related health inequality in mothers and children using the concentration index. Epidemiol Biostat Public Health.

[9] Moazzeni MS (2013). Maternal mortality in the Islamic Republic of Iran: on track and in transition. Matern Child Health J.

[10] Aghajanian A, Merhyar AH (1999). Fertility, contraceptive use and family planning program activity in the Islamic Republic of Iran. Int Fam Plan Perspect.

[11] Hogan MC, Foreman KJ, Naghavi M, Ahn SY, Wang M, Makela SM (2010). Maternal mortality for 181 countries, 1980-2008: a systematic analysis of progress towards Millennium Development Goal 5. Lancet.

[12] Khlat M, Ronsmans C (2000). Deaths attributable to childbearing in Matlab, Bangladesh: indirect causes of maternal mortality questioned. Am J Epidemiol.

[13] Hamadan University of Medical Sciences Health network development. http://mboh.umsha.ac.ir/.

[14] Centers for Disease Control and Prevention (2014). Preterm birth. http://www.cdc.gov/reproductivehealth/MaternalInfantHealth/PretermBirth.htm.

[15] World Health Organization 10 facts on obesity. http://www.who.int/features/factfiles/obesity/facts/en/.

[16] Cham M, Sundby J, Vangen S (2005). Maternal mortality in the rural Gambia, a qualitative study on access to emergency obstetric care. Reprod Health.

[17] National Institute of Health (2013). What are some common complications of pregnancy?. http://www.nichd.nih.gov/health/topics/pregnancy/conditioninfo/Pages/complications.aspx.

[18] Ramos S, Karolinski A, Romero M, Mercer R, Maternal Mortality in Argentina Study Group (2007). A comprehensive assessment of maternal deaths in Argentina: translating multicentre collaborative research into action. Bull World Health Organ.

[19] Conde-Agudelo A, Belizán JM (2000). Maternal morbidity and mortality associated with interpregnancy interval: cross sectional study. BMJ.

[20] Liu S, Liston RM, Joseph KS, Heaman M, Sauve R, Kramer MS (2007). Maternal mortality and severe morbidity associated associated with low-risk planned cesarean delivery versus planned vaginal delivery at term. CMAJ.

[21] Deneux-Tharaux C, Carmona E, Bouvier-Colle MH, Bréart G (2006). Postpartum maternal mortality and cesarean delivery. Obstet Gynecol.

[22] Conde-Agudelo A, Belizán JM, Lindmark G (2000). Maternal morbidity and mortality associated with multiple gestations. Obstet Gynecol.

